# Double-Emulsion Perfluorocarbon Nanodroplets for Ultrasound and Photoacoustic Image-Guided Drug Delivery and Release

**DOI:** 10.1021/acsnano.5c12290

**Published:** 2025-12-09

**Authors:** Euisuk Chung, Andrew X. Zhao, Stanislav Y. Emelianov

**Affiliations:** † School of Electrical and Computer Engineering, 1372 Georgia Institute of Technology, Atlanta, Georgia 30332, United States; ‡ Wallace H. Coulter Department of Biomedical Engineering, Georgia Institute of Technology and Emory University School of Medicine, Atlanta, Georgia 30332, United States

**Keywords:** double-emulsion perfluorocarbon nanodroplets, image-guided drug delivery, focused ultrasound, ultrasound-responsive drug release, ultrasound/photoacoustic imaging

## Abstract

Phase-change perfluorocarbon nanodroplets (PFCnDs) have shown potential for controlled, image-guided drug delivery. However, their clinical translation is limited by the poor encapsulation of hydrophilic therapeutics and unintended cargo release during ultrasound (US) or photoacoustic (PA) imaging. In this study, we present the development of double-emulsion perfluorocarbon nanodroplets (dePFCnDs) designed to encapsulate hydrophilic payloads while enabling efficient, real-time, focused ultrasound (FUS) triggered release. Cryo-transmission electron microscopy revealed that the dePFCnDs consist of a hydrophilic inner core surrounded by a perfluorocarbon layer that supports US/PA imaging. Compared to conventional single-emulsion PFCnDs, the double-emulsion structure significantly enhanced the loading efficiency tested using the fluorescent hydrophilic model drug, calcein. Furthermore, improved stability was demonstrated showing minimal calcein leakage under imaging conditions. Release studies demonstrated selective responsiveness of dePFCnDs to FUS stimulation, with negligible response to thermal or laser triggers. Optimizing focused ultrasound parameters further enhances release efficiency, enabling precise spatial and temporal control. In vitro and in vivo experiments confirmed the feasibility of utilizing real-time US/PA tracking of droplet localization, and changes in US/PA signal as a proxy for payload release. This proof-of-concept study demonstrates the potential of dePFCnDs as a hydrophilic therapeutics carrier that provides a robust, safe, and effective platform for ultrasound-mediated, image-guided delivery and release.

## Graphical abstract

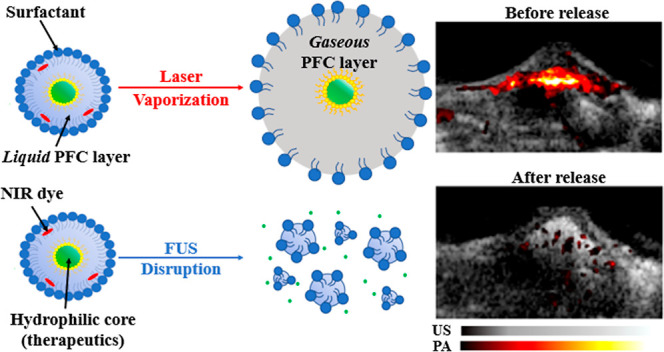

## Introduction

One of the major challenges in drug delivery and controlled release is minimizing off-target effects while maximizing therapeutic efficacy. Systemically administered drugs, such as those delivered by injection or oral administration, often disperse throughout the body, with only a small fraction reaching the intended target.^1–3^ For example, in chemotherapy, less than 1% of the administered dose typically accumulates in solid tumors, while the remainder is distributed to healthy tissues, causing severe side effects such as cardiac toxicity, bone marrow suppression, and peripheral neuropathy.^4^ This off-target distribution is not limited to chemotherapeutics but is also a critical issue for other drug classes, including anti-inflammatory agents, antibiotics, and emerging biologics such as peptide and RNA-based therapeutics.^5–7^

Stimuli-responsive drug delivery systems have emerged as promising strategies to address these limitations. These systems are designed to prevent the off-target drug release while enabling controlled release in response to specific internal or external stimuli. Carriers triggered by endogenous factors, such as acidic pH, elevated glutathione, or enzyme overexpression, offer selective release but often lack precise spatiotemporal control.^8–12^ In contrast, systems that respond to exogenous stimuli, including heat, magnetic fields, light, and ultrasound (US), can achieve highly localized and temporally controlled drug release. However, the use of exogenous triggers typically requires real-time imaging to guide stimulus application, confirm carrier localization, and monitor therapeutic response.^13–18^ Among these external modalities, focused ultrasound (FUS) stimulation offers various advantages, including deep tissue penetration, precise spatial control, nonionization, and compatibility with real-time feedback.^19–21^ When integrated with US and photoacoustic (PA) imaging, FUS-based systems enable real-time visualization of carrier distribution and drug release, which is particularly valuable for precision medicine.^22^

Phase-change perfluorocarbon nanodroplets (PFCnDs) are widely studied ultrasound-responsive drug carriers.^23^ PFCnDs consist of a perfluorocarbon core, such as perfluoropentane or perfluorohexane, stabilized by a lipid or surfactant shell. Upon exposure to laser irradiation or high-intensity FUS, the core vaporizes, generating echogenic microbubbles that simultaneously facilitate drug release and enhance US contrast.^24,25^ When optical absorbers are incorporated into the shell, PFCnDs also provide strong PA signals, enabling dual-mode US/PA imaging and real-time monitoring of droplet behavior.^26,27^ Furthermore, combining acoustic and optical activation can enhance vaporization efficiency, allowing controlled drug release at reduced energy levels.

Despite these advantages, conventional single-emulsion PFCnDs have notable limitations. Their hydrophobic cores restrict efficient loading of hydrophilic therapeutics, such as small-molecule kinase inhibitors, peptide therapeutics, and genome-editing ribonucleoproteins. In addition, unintended leakage or premature vaporization during laser and ultrasound exposure for imaging can compromise treatment specificity and safety.^23,28–30^

To overcome these challenges, researchers have explored double-emulsion PFC nanodroplets (dePFCnDs), which feature an internal hydrophilic core capable of encapsulating hydrophilic drugs. Early versions of dePFCnDs demonstrated ultrasound-triggered release but exhibited large particle sizes and poor stability, limiting tissue penetration and increasing the risk of cargo leakage.^31,32^ More recent formulations achieved submicron sizes but still faced issues with unintended release under imaging conditions.^33^

In this study, we present an optimized double-emulsion PFCnDs specifically engineered for controlled delivery of hydrophilic therapeutics with image guidance (Figure 1). We systematically characterize the nanodroplets in terms of size distribution, encapsulation efficiency, stability, and ultrasound-triggered release. Additionally, using combined US/PA imaging, real-time tracking of dePFCnDs delivery, and hydrophilic cargo release estimation in both in vitro and in vivo settings. The engineered dePFCnDs offer a promising strategy for precisely controlled image-guided drug delivery and release.

**Figure 1:**
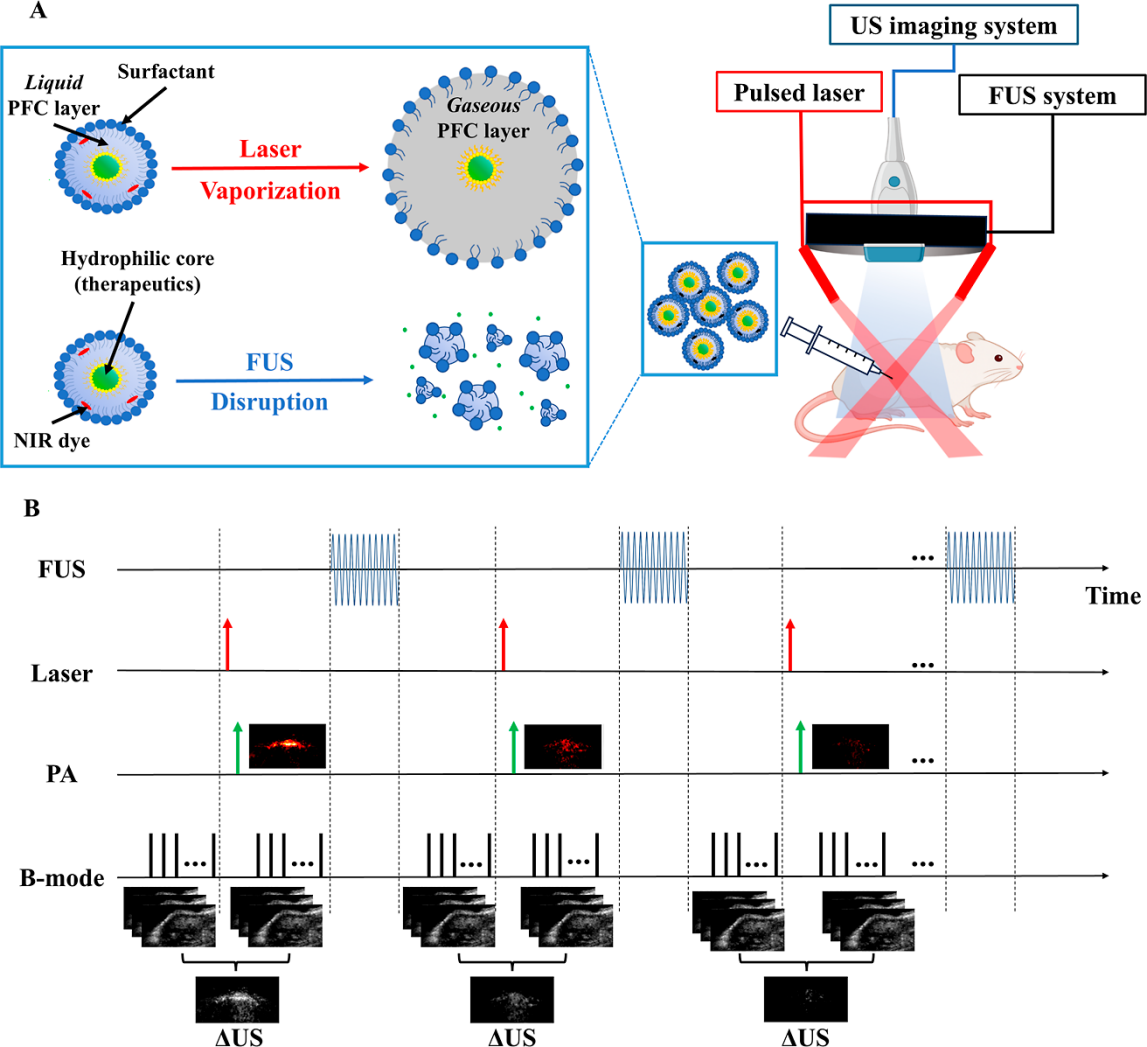
Experiment setup and US/PA/FUS sequence for dePFCnDs imaging and encapsulated cargo release. (A) Double-emulsion phase-changing nanodroplets (dePFCnDs) for US/PA imaging, and FUS-mediated encapsulated cargo release. (B) Sequence for dePFCnDs US/PA imaging and FUS-mediated encapsulated cargo release.

## Results and Discussion

Double-emulsion perfluorocarbon nanodroplets (dePFCnDs) were synthesized and characterized to enable encapsulation and controlled delivery and release of a model drug (hydrophilic fluorescent dye calcein). Cryo-transmission electron microscopy (cryo-TEM) confirmed the presence of an internal hydrophilic core surrounded by a perfluorocarbon (PFC) layer, which structurally distinguishes them from conventional single-emulsion PFC nanodroplets (PFCnDs) that lack this hydrophilic core (Figure 2A). Dynamic light scattering (DLS) measurements showed that dePFCnDs had an average diameter of approximately 320 nm (Figure 2B), significantly smaller than conventional PFCnDs (∼480 nm), and within the tumor pore cutoff range of 400–600 nm. This size reduction is expected to improve tumor accumulation via the enhanced permeability and retention (EPR) effect.^34^

**Figure 2:**
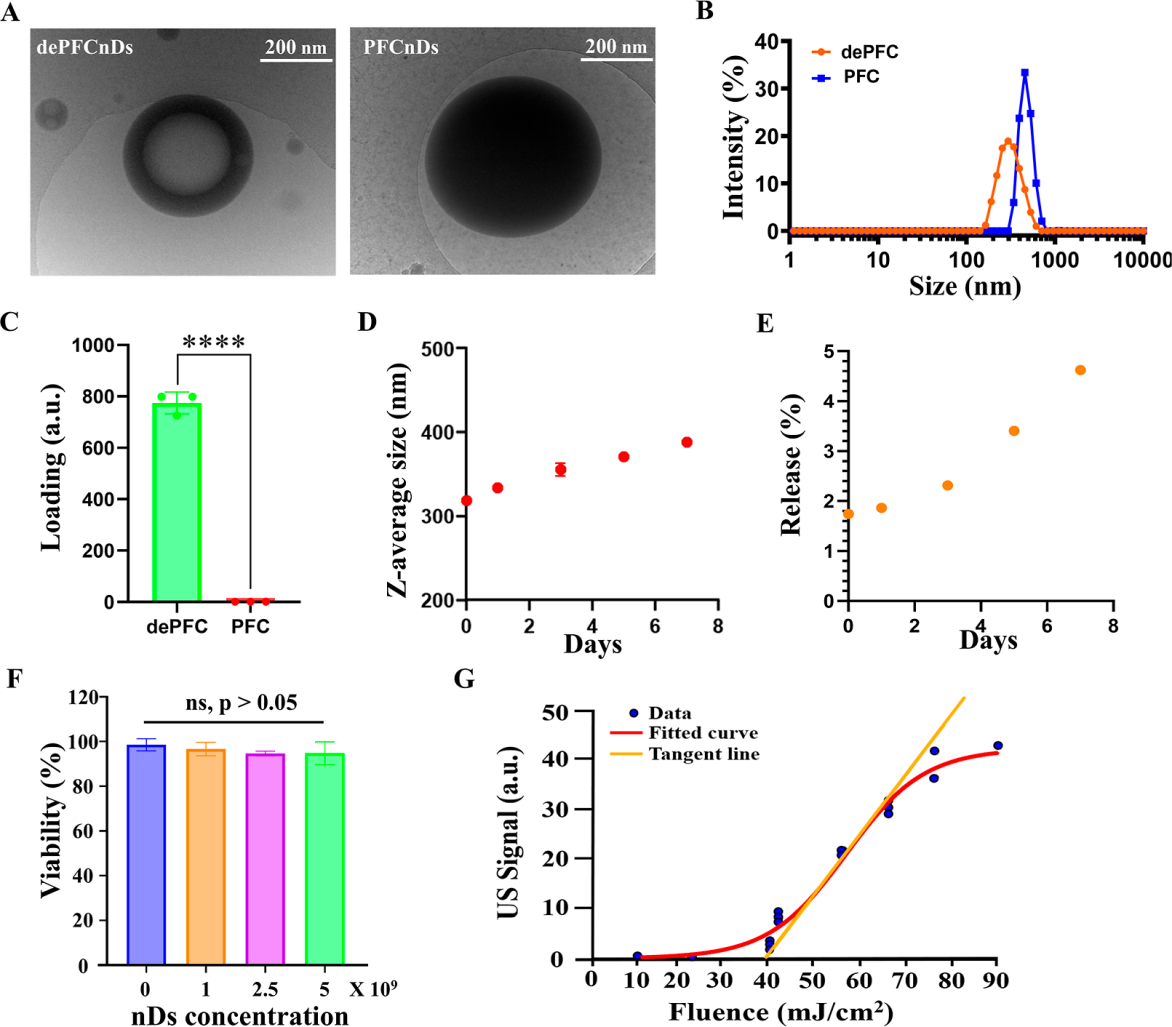
Characterization of dePFCnDs. (A) Representative cryo-TEM images comparing the internal structures of dePFCnDs and conventional single-emulsion PFCnDs. Scale bar = 200 nm. (B) Size distribution profiles of dePFCnDs and PFCnDs measured by DLS. (C) Loading efficiency comparison between dePFCnDs and PFCnDs quantified by fluorescence intensity of encapsulated hydrophilic fluorescent dye (calcein). Data are presented as mean ± SD (*n* = 3, a.u. = arbitrary units, ****p < 0.001). (D) Stability of dePFCnDs was demonstrated by monitoring the change in z-average size over 7 days (*n* = 3; data are presented as mean ± SD). (E) Leakage of calcein from the dePFCnDs, represented as the percentage of the initially encapsulated amount over 7 days (*n* = 3, data are presented as mean ± SD). (F) Cytotoxicity assessment of dePFCnDs via MTT assay, evaluating the viability of RAW 264.7 cells exposed to varying concentrations of dePFCnDs. No significant differences observed among groups (*n* = 4; data are presented as mean ± SD; ns = nonsignificant (*p* > 0.05), one-way ANOVA with Tukey’s multiple comparison test). (G) Vaporization threshold analysis of dePFCnDs, illustrating changes in ultrasound signals with increasing laser fluence.

The encapsulation efficiency was enhanced in dePFCnDs due to the hydrophilic core, as verified by fluorescence-based quantification using calcein as a model drug (Figure 2C). Stability assessments over 7 days showed minimal size increase (<100 nm) and less than 5% leakage (Figure 2D,E), indicating that the dePFCnDs maintain structural integrity over clinically relevant time scales. Cytotoxicity evaluation via MTT assays demonstrated that dePFCnDs exhibited low toxicity, with cell viability exceeding 90% across tested concentrations (Figure 2F). The vaporization threshold was approximately 40 mJ/cm^2^, which is compatible with safe and efficient vaporization, producing detectable US signals (Figure 2G).

To evaluate the stimulus specificity of dePFCnDs, release profiles were assessed under heating (60 °C for 10 min), laser irradiation (70 mJ/cm^2^, 100 pulses), and focused ultrasound (FUS; 2.16 MPa, 50% duty cycle, 300 ms pulse repetition interval (PRI), 120 s total sonication). Heating and laser exposure each induced negligible calcein release (<2%), which was not significantly different from that of unstimulated controls (Figure 3A). In contrast, FUS stimulation triggered encapsulated cargo release exceeding 60%, demonstrating selective ultrasound responsiveness. Additional experiments varying temperature (25–70 °C) and laser fluence confirmed that thermal effects alone or laser exposure did not significantly contribute to encapsulated cargo release (Figure 3B–D), supporting the conclusion that dePFCnDs selectively release the encapsulated payload by acoustic stimuli and the suitability for safe, image-guided drug delivery with the ANSI safety limits for PA imaging.^35–38^

**Figure 3:**
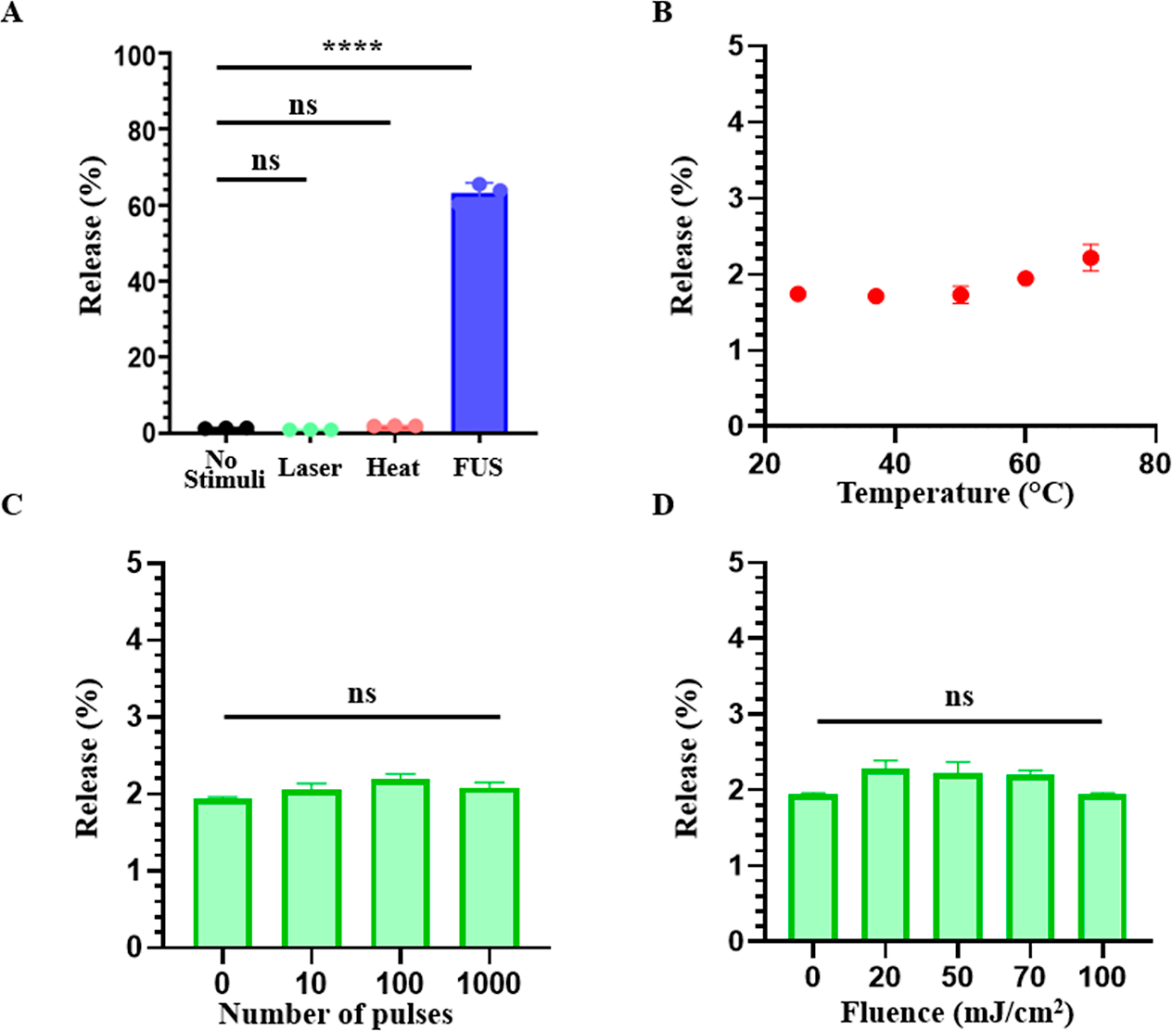
Stimulus-responsive release from dePFCnDs. (A) Comparison of encapsulated calcein release efficiency from dePFCnDs under various stimulation conditions: control (no stimulus), laser irradiation (100 pulses, 70 mJ/cm^2^), heat treatment (60 °C, 10 min), and focused ultrasound (2.16 MPa, 50% duty cycle, 300 ms PRI, 2 min). Significant release observed exclusively under FUS stimulation (****p < 0.0001, ns = nonsignificant vs control) (B) Evaluation of thermal sensitivity of calcein release from dePFCnDs incubated at various temperatures (25, 37, 50, 60, and 70 °C) for 10 min. (C) Effect of the number of laser pulses (0, 10, 100, and 1000 pulses at a fixed fluence of 70 mJ/cm^2^) on the release efficiency of calcein from dePFCnDs. No significant differences were observed among groups. (D) Release efficiency of dePFCnDs at various fluences (0, 20, 50, 70, and 100 mJ/cm^2^) at 100 laser pulses. No significant differences were observed among the groups (*n* = 3 for all experiments; data are presented as mean ± SD). Statistical significance was determined using one-way ANOVA with Tukey’s multiple comparison test.

After validating the encapsulation and release of the model drug (calcein), we evaluated dePFCnDs with poly I:C, a clinically investigated nucleic acid payload, to assess their applicability beyond low-molecular-weight dyes. Poly I:C is a double-stranded RNA mimetic recognized by Toll-like receptor 3 (TLR3), inducing downstream activation of NF-κB, upregulation of inducible nitric oxide synthase, and nitric oxide production.^39^ Macrophage-derived nitrite was quantified using the Griess assay. Following the two probe-ultrasonication steps required for dePFCnD formation, poly I:C activity was confirmed by Griess analysis, indicating that the brief sonication and the double-stranded structure of poly I:C preserved molecular integrity during formulation (Figure 4A).

**Figure 4:**
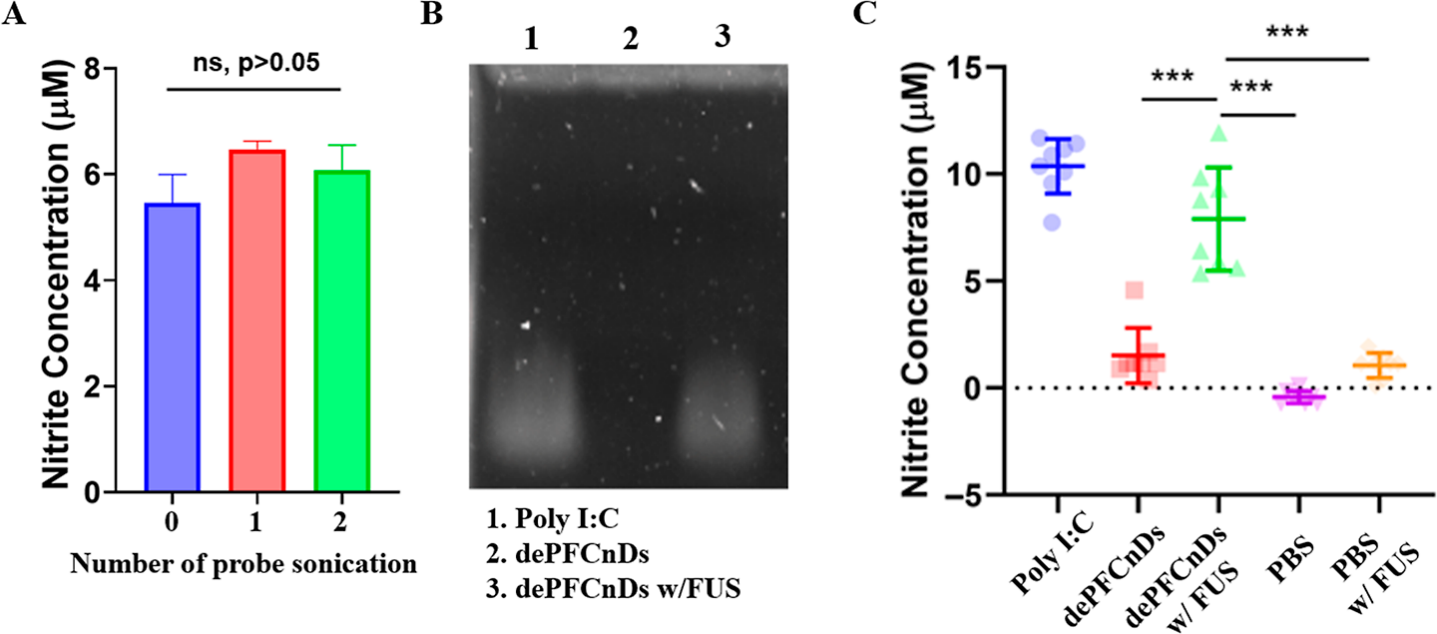
Poly I:C encapsulation to dePFCnDs and release from dePFCnDs. (A) The activity of poly I:C remains unchanged after the two probe sonication steps needed to produce dePFCnDs, as indicated in the Griess assay (Data represent mean ± SD, *n* = 3, ns represents not significant (*p* > 0.05)). (B) Agarose gel electrophoresis of poly I:C loaded into dePFCnDs and subjected to FUS (PNP: 2.16 MPa, PRI: 15 ms, duration 5 min) in comparison to unprocessed poly I:C. The released poly I:C shows no signs of degradation. (C) Nitrite generated from the RAW 264.7 macrophages that were incubated with dePFCnDs and exposed to FUS (PNP: 1.08 MPa, PRI: 15 ms, duration: 5 min) with mean ± SD of *n* = 8 replicates (Tukey’s multiple comparisons test, *p* < 0.001 represented by ***).

Encapsulation and FUS-triggered release of poly I:C were evaluated by agarose gel electrophoresis. The poly I:C–loaded dePFCnDs without FUS exposure showed no detectable bands, whereas bands were observed from FUS-exposed dePFCnDs, confirming negligible leakage and selective release (Figure 4B). These results also indicate that poly I:C is not fragmented or destroyed during the release process by FUS and dePFCnD fragments do not inhibit poly I:C activity.

To simulate a more realistic scenario, RAW 264.7 macrophages were seeded in film-bottom plates and incubated with poly I:C loaded dePFCnDs. Wells exposed to FUS exhibited increased nitrite production compared with dePFCnDs alone, PBS, or PBS + FUS (Figure 4C). These findings provide evidence that nucleic-acid payload can be encapsulated within dePFCnDs and delivered to cells without compromising activity.

Stimulus-responsive release results showed that the encapsulated cargo can be released selectively by FUS. To optimize the FUS-mediated release process, FUS parameters were systematically varied. Increased peak negative pressure (PNP), extended sonication duration, and higher duty cycles all enhanced release efficiency, which plateaued after approximately 300 s of total sonication (Figure 5A–C). Additionally, PRI optimization revealed that a pulse-off time of ∼8 ms is optimal for maximizing release efficiency. Longer PRIs allowed partially vaporized droplets to recondense before the next pulse, reducing release efficiency (Figure 5D). And too short PRIs resulted in insufficient pulse on time to disrupt dePFCnDs for encapsulated payload release. These findings highlight the importance of precisely controlling sonication parameters to achieve efficient and reproducible release.

**Figure 5:**
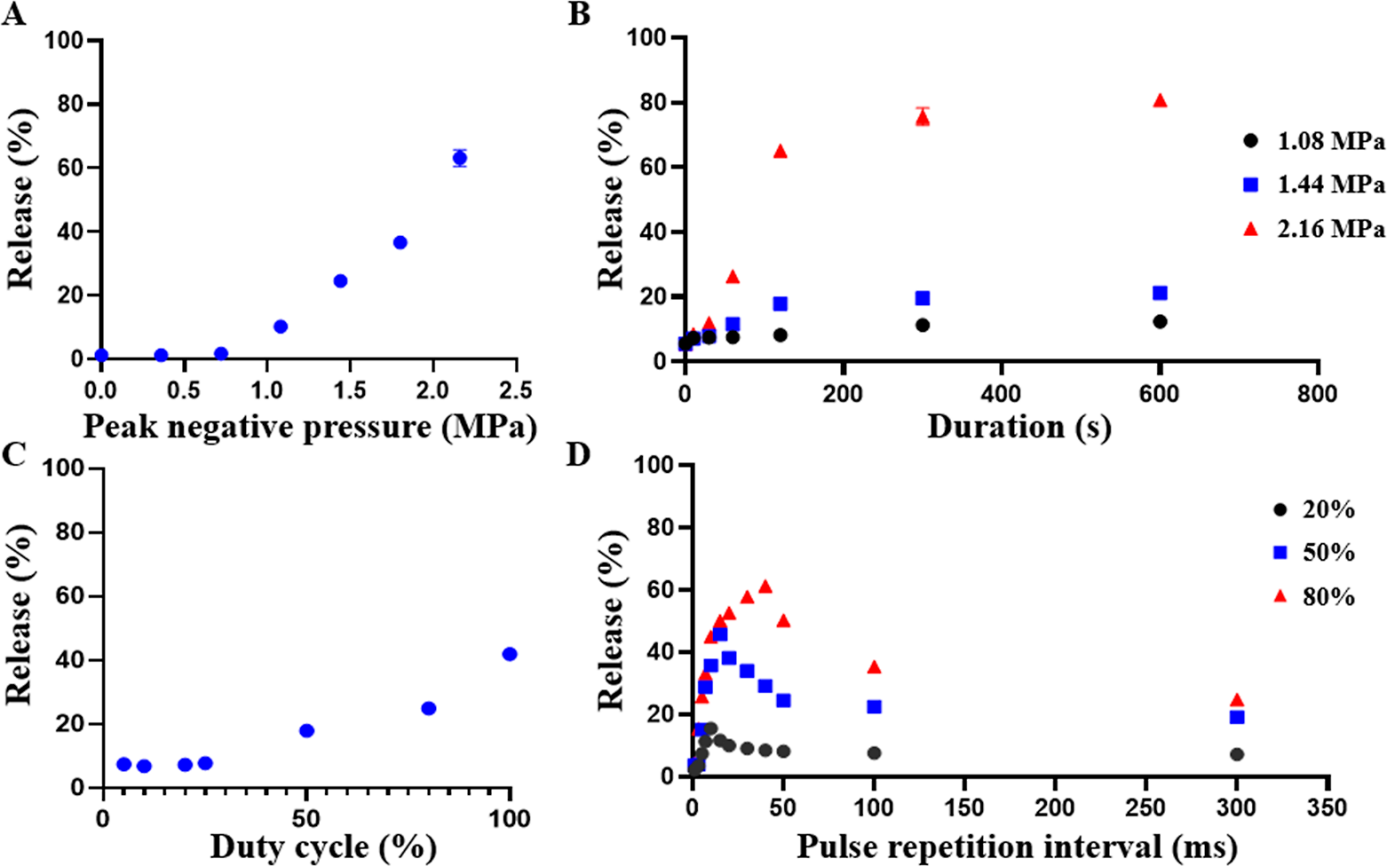
The release efficiency of dePFCnDs as a function of focused ultrasound parameters. (A) Effect of ultrasound peak negative pressure (PNP; 0–2.16 MPa) on calcein release efficiency from dePFCnDs (300 ms PRI, 50% duty cycle, 2 min), demonstrating a monotonic increase. (B) Influence of sonication duration (0–600 s) on release efficiency at different PNPs (1.08, 1.44, and 2.16 MPa; 50% duty cycle, 300 ms PRI). (C) Effect of varying duty cycle (5–100%) on release efficiency, demonstrating a monotonic increase (1.44 MPa PNP, 300 ms PRI, 2 min). (D) Impact pulse repetition interval (PRI, 1–300 ms) on release efficiency at different duty cycles (20, 50, and 80%; 1.44 MPa PNP, 2 min). Data are presented as mean ± SD (*n* = 3 for all experiments).

Cavitation signal spectra for PBS and nanodroplet state dePFCnDs were indistinguishable, exhibiting neither harmonic nor broadband signals. After laser-induced vaporization of the PFC core, short-pulse FUS sonication produced harmonics, consistent with stable cavitation. Under long-pulse FUS sonication of vaporized droplets, spectra exhibited concurrent harmonic features with an elevated broadband signal, consistent with inertial cavitation and droplet disruption (Figure 6A). Together with release kinetics, the data support a two-step mechanism: optical or acoustic droplet vaporization followed by mechanical disruption of the nanodroplets to release the encapsulated cargo. From these results, the spectral analysis of the cavitation signals can be used to adjust the FUS parameters for safe and efficient delivery.

**Figure 6:**
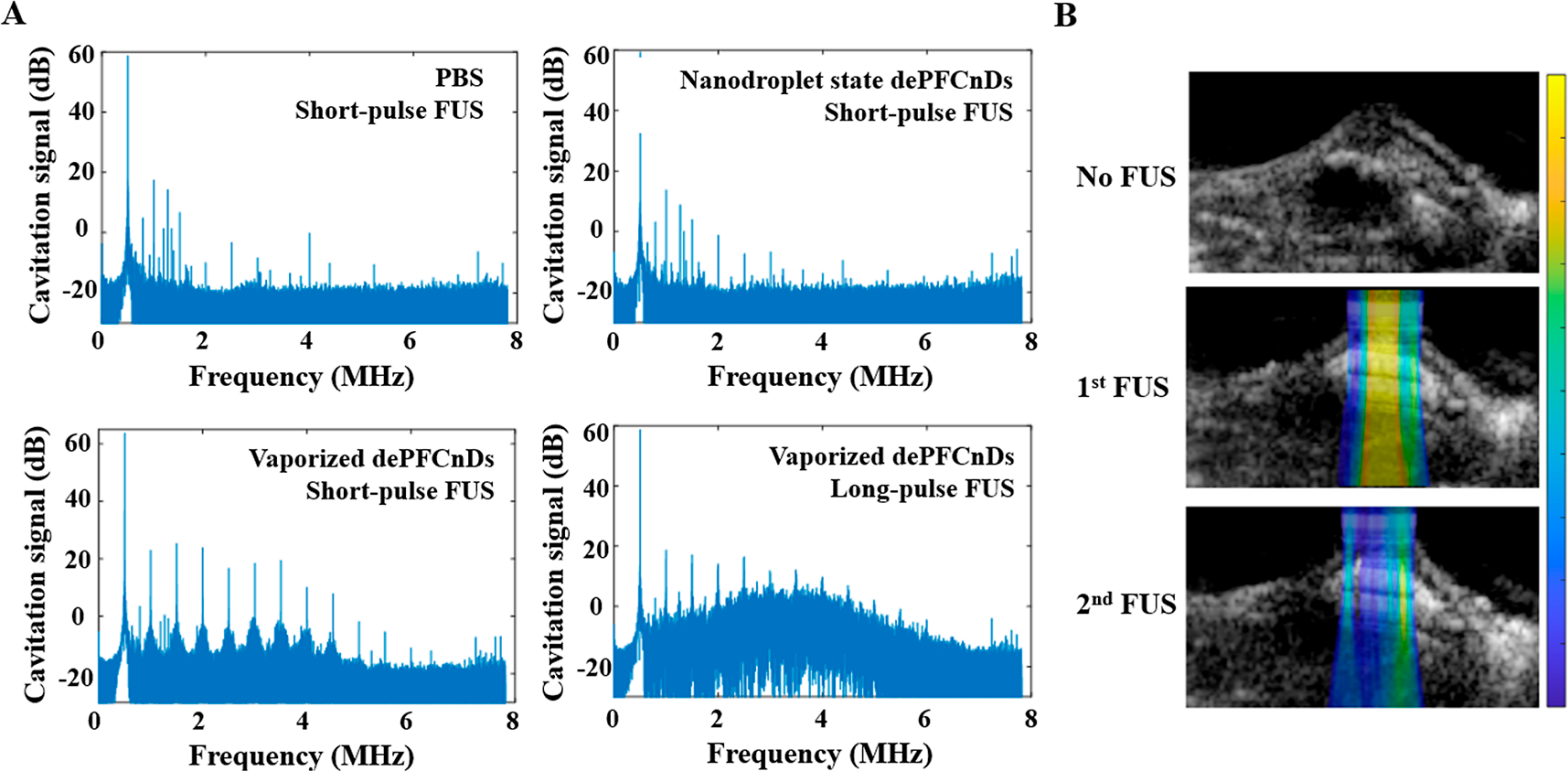
Bubble dynamics of dePFCnDs during FUS stimulation. (A) Spectra of the cavitation signals under four conditions: PBS control with short-pulse FUS; nanodroplet-state (prevaporized) dePFCnDs with short-pulse FUS; vaporized dePFCnDs after laser activation with short-pulse FUS showing harmonics consistent with stable cavitation; vaporized dePFCnDs with long-pulse FUS showing harmonics and broadband signal consistent with inertial cavitation and droplet disruption. (B) In vivo acoustic cavitation mapping overlaid in the B-mode US image provides the locations and intensities of the vaporized dePFCnDs during FUS.

In vivo acoustic cavitation mapping overlaid in the B-mode US image provides the location and intensity of the activated dePFCnDs during FUS (Figure 6B). Post-FUS stimulation, cavitation signals decrease, enabling the estimation of release efficiency. Using cavitation maps to guide FUS dosing maximizes on-target release while minimizing off-target exposure.

A key advantage of the dePFCnDs is their stability under photoacoustic (PA) imaging conditions. Unlike conventional nanodroplets, the dePFCnDs did not exhibit unintended leakage during PA imaging,^31,33,40^ allowing for nondestructive, real-time tracking of nanodroplet delivery. Integrated ultrasound (US) and PA imaging demonstrated that PA and differential US (ΔUS) signals decreased monotonically with increasing release fractions, establishing a relationship between signal intensity and encapsulated payload release (Figure 7A–C). This enables real-time estimation of both nanodroplet concentration and local release during therapy, enhancing the potential for image-guided precision drug delivery and release.

**Figure 7:**
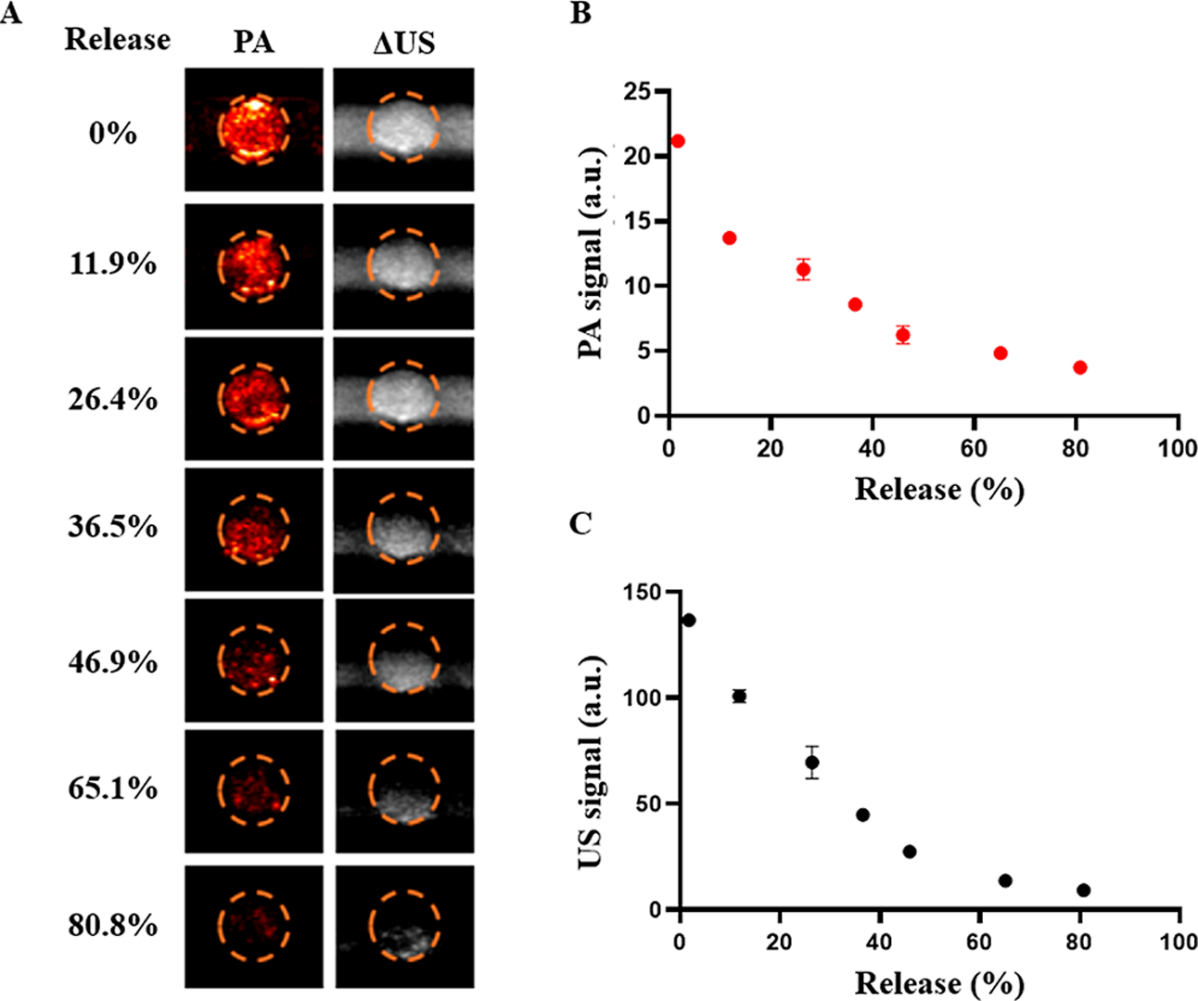
In vitro nanodroplet tracking and release monitoring of dePFCnDs using combined US/PA imaging. (A) Representative PA and differential US (ΔUS) images of dePFCnDs after FUS exposure of varying conditions, each labeled with the corresponding release percentages. Orange dashed circles indicate the region of interest. Ultrasound signals outside the dashed ROIs arise from single-angle plane-wave side lobes and grating lobes (large point spread function), not from droplet diffusion or deposition. Only signals within the ROI area, excluding the side lobe and grating region, were quantified. (B) Correlation of the integrated PA signal of dePFCnDs with known release percentages. (C) Correlation of integrated differential US intensity of the dePFCnDs with known release levels. Data represent mean ± SD (*n* = 3 for all experiments).

In vivo studies confirmed the feasibility of ultrasound-guided hydrophilic payload delivery using dePFCnDs. Following intratumoral injection, dePFCnDs produced strong differential US and PA signals, confirming successful localization at the target site. Subsequent FUS exposure resulted in a substantial PA and differential US signal reduction. These signal reductions served as proxies for nanodroplet disruption and for the release of the encapsulated hydrophilic payload (Figures 8 and 9). Two different FUS intensities were evaluated to demonstrate therapeutic versatility. Low-intensity FUS (1.08 MPa) selectively triggered cargo release without significant thermal effects, while high-intensity FUS (2.16 MPa) induced bubble formation and thermal ablation, enabling combined mechanical and thermal tumor therapy.^41^ These results illustrate the adaptability of dePFCnDs for both nonablative release and ablative therapeutic strategies within a single platform.

**Figure 8:**
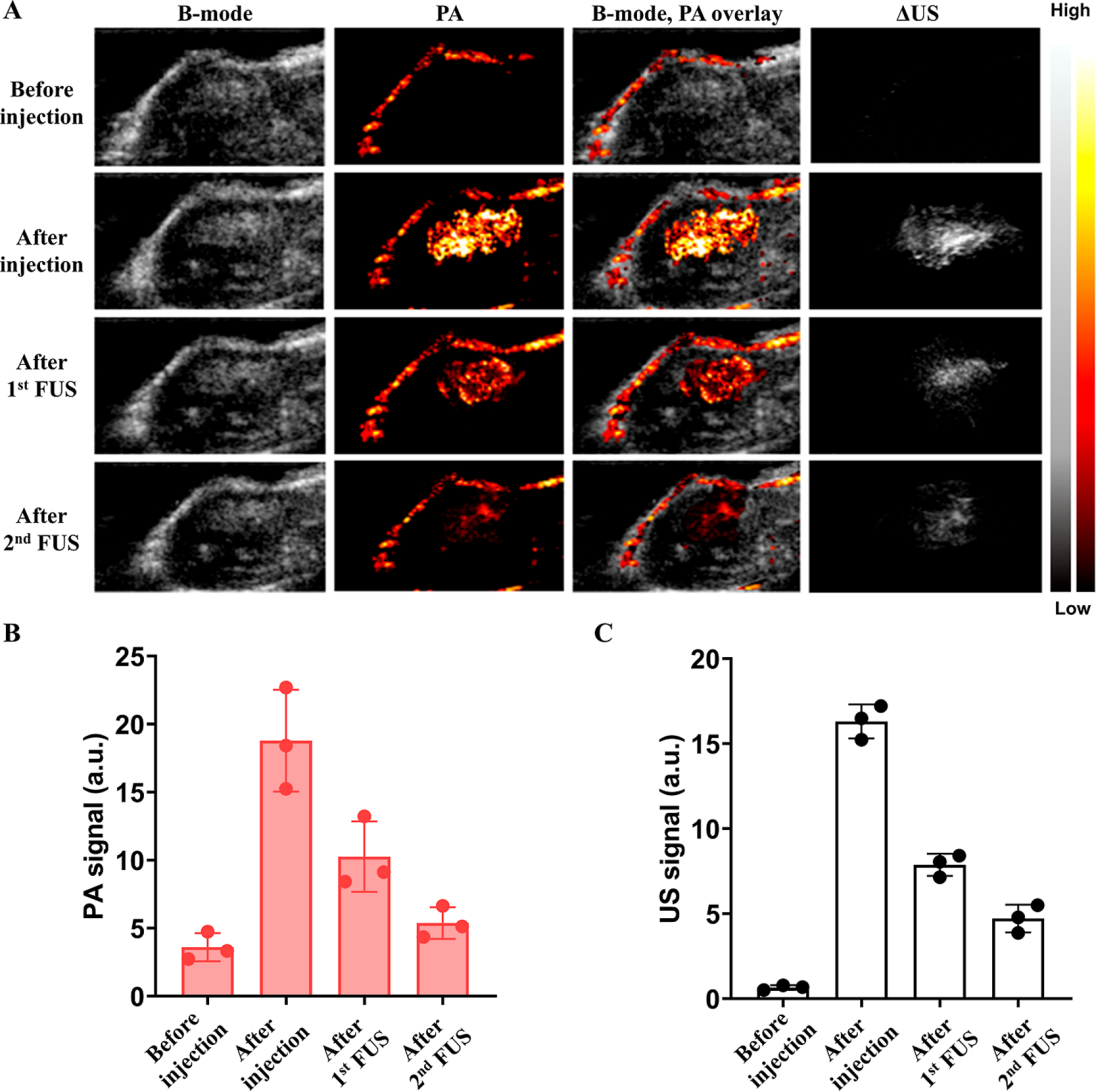
In vivo US/PA images of dePFCnDs with low intensity FUS-mediated payload release (1.08 MPa PNP, 50% duty cycle, 15 ms PRI, 300 s duration). (A) Images are presented in four columns: B-mode ultrasound (left), photoacoustic (PA) images (second column), B-mode/PA overlay images (third column), and differential ultrasound (ΔUS) images (right). Images were acquired at four stages: (1) before injection, (2) immediately after dePFCnDs injection, (3) following the first low-intensity (1.08 MPa) FUS sonication, and (4) after the second low-intensity (1.08 MPa) FUS sonication. (B) PA signal of the dePFCnDs, (C) Differential US intensity of the dePFCnDs before injection, after injection, after the first low-intensity (1.08 MPa) FUS sonication, and after the second low-intensity (1.08 MPa) FUS sonication (*n* = 3 for all experiments).

**Figure 9:**
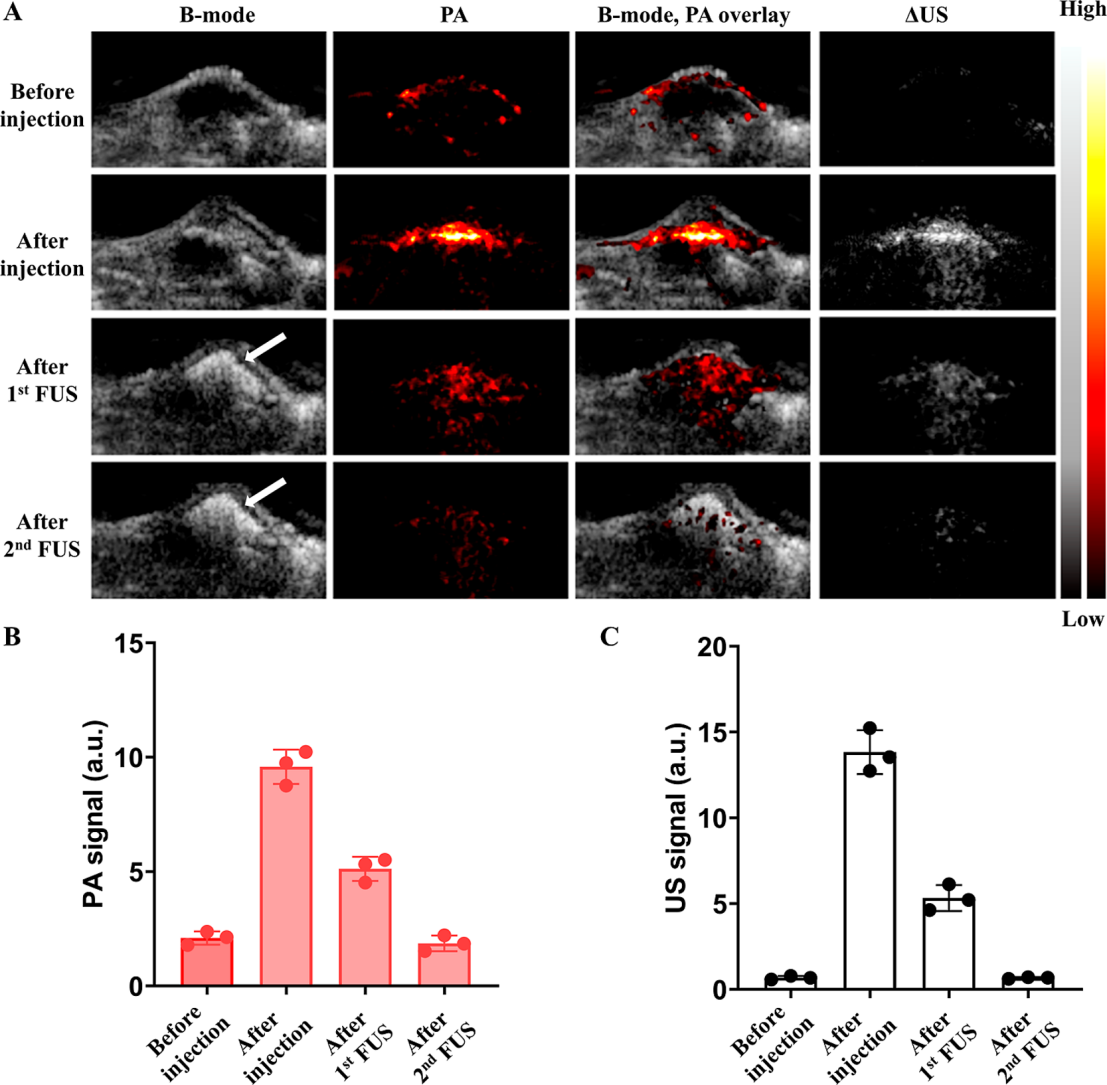
In-vivo US/PA images of dePFCnDs with high intensity FUS-mediated payload release (2.16 MPa PNP, 50% duty cycle, 15 ms PRI, 300 s duration). (A) Images are presented in four columns: B-mode ultrasound (left), photoacoustic (PA) images (second column), B-mode/PA overlay images (third column), and differential ultrasound (ΔUS) images­(right). Images were acquired at four stages: (1) before injection, (2) immediately after dePFCnDs injection, (3) following the first high-intensity (2.16 MPa) FUS sonication, and (4) after the second high-intensity (2.16 MPa) FUS sonication. White arrows (third row, fourth row B-mode US images) indicate regions with hyperechoic area resulting from thermal ablation. (B) PA signal of the dePFCnDs. (C) Differential US intensity of the dePFCnDs before injection, after injection, after the first high-intensity (2.16 MPa) FUS sonication, and after the second high-intensity (2.16 MPa) FUS sonication (*n* = 3 for all experiments).

However, release efficiency plateaued at approximately 80% and was not strictly monotonic across repeated FUS exposures. This may be attributed to size heterogeneity among droplets, as smaller droplets likely require higher acoustic pressures to vaporize and may remain intact under standard exposure conditions.^25,42^ Additionally, probe sonication used in droplet synthesis may contribute to this heterogeneity, complicating precise drug dosing and potentially affecting in vivo biodistribution. Future efforts employing microfluidic or extrusion-based manufacturing could improve droplet uniformity, enhancing encapsulated therapeutics release consistency and therapeutic reproducibility.^32,43,44^

While initial cytotoxicity assessments confirmed good biocompatibility, long-term studies evaluating the biodistribution of fluorinated components, systemic clearance pathways, and potential immune responses are essential before clinical translation. A thorough investigation of these safety profiles will be critical to advancing dePFCnDs toward clinical application.^45^

The dePFCnDs offer selective, ultrasound-triggered release of hydrophilic therapeutics, along with image-guided monitoring capabilities. This image-guided delivery and release platform addresses key limitations of conventional nanodroplet-based hydrophilic therapeutics delivery and release systems, holding significant promise for precision release.

## Conclusion

This study demonstrated that double-emulsion perfluorocarbon nanodroplets (dePFCnDs) offer a promising approach to ultrasound-mediated drug delivery and release, with noninvasive, real-time monitoring and precise control over drug release. The developed dePFCnDs enable minimal payload leakage, improved hydrophilic cargo loading efficiency compared to conventional single-emulsion PFCnDs, and selective ultrasound responsiveness while allowing for the simultaneous tracking and estimation of encapsulated payload release during FUS. Moreover, the ability to integrate mechanical, thermal, and chemical therapeutic effects within a single treatment session highlights the versatility of this approach for clinical applications. Future studies should focus on improving particle size uniformity, thoroughly evaluating long-term biocompatibility and bioelimination pathways, and exploring combination therapies, including immunotherapy, to enhance both the safety and therapeutic efficacy of dePFCnDs for clinical translation.

## Methods and Experimental

### Materials

008-FluoroSurfactant (RAN Biotechnologies), perfluorohexane (PFH, FluoroMed), calcelin (Sigma-Aldrich), phosphate buffered saline (PBS, Sigma-Aldrich), pluronic f-68 (Sigma-Aldrich), Epolight 3072 (Epoline), 3-(4,5-dimethylth-iazol-2-yl)-2,5-diphenyltetrazolium bromide (MTT, EMD Millipore), dimethyl sulfoxide (DMSO, Sigma-Aldrich), polyacrylamide solution (Sigma-Aldrich), ammonium sulfate solution (APS, Sigma-Aldrich), and N, N, *N*′,*N*′-Tetramethylethylenediamine­(TEMED, Sigma-Aldrich) were used by manufacturer’s instructions.

### Synthesis of dePFCnDs

The dePFCnDs were synthesized using a probe sonication method. First, 75 mg of 008-FluoroSurfactant (RAN Biotechnologies, MA, USA) was added to 1.5 mL of ice-cold perfluorohexane (PFH, FluoroMed, TX, USA). Then, 1.5 mL of a hydrophilic model drug solution (0.5 mg/mL calcein (Sigma-Aldrich, MO, USA) in PBS, filtered through a 0.2 μm filter) was mixed with the 008-FluoroSurfactant in PFH. This mixture was probe sonicated (Q700, QSONICA, CT, USA) in an ice bath at an amplitude of 1 (30 W/cm^2^) for 30 s to fabricate a stable primary emulsion. The first emulsion (1 mL) was added to the 3 mL of 0.5% pluronic f-68 (20-times dilute 10% pluronic f-68 (Sigma-Aldrich, MO, USA) in PBS) with NIR dye (0.2 mg of Epolight 3072 (Epolin, NJ, USA)) to maximize absorbance at 1064 nm and sonicated for 60s with the same amplitude as the first emulsion to synthesize dePFCnDs.

The dePFCnDs mixture was centrifuged at 43 RCF for 3 min using a mini-centrifuge (5452 minispin Centrifuge; Eppendorf, Hamburg, Germany) to remove large particles and unencapsulated dyes. After discarding the pellet, the supernatant was centrifuged 3 times at 4500 RCF for 3 min to remove the residual free hydrophilic payload. For each centrifugation step, the supernatant was discarded, and the pellet was resuspended in 4 mL of PBS.

### Characterization of dePFCnDs

The average dePFCnD size was determined using a Malvern Zetasizer Dynamic light scattering (DLS, Zetasizer Nano ZS; Malvern Panalytical, Malvern, UK) after a 1000-fold dilution to ensure the concentration was sufficiently low.

The internal structure of the dePFCnDs was verified utilizing cryo-transmission electron microscopy (TEM) imaging. The dePFCnDs were plunge-frozen onto glow-discharged, 200-mesh copper Quantifoil grids (Quantifoil, Germany) in liquid ethane using a Vitrobot Mark IV (ThermoFisher, Hillsboro, OR, USA). Cryo-TEM images were acquired using a 120 kV Talos L120C TEM (Thermo Fisher Scientific, USA) with a Ceta CMOS camera. The dose rates were between 15 and 20 electrons/Å2 at magnifications of 36 k (pixel size of 4 Å/pixel) and 45 k (pixel size of 3.2 Å/pixel).

To evaluate the loading efficiency of hydrophilic therapeutics, the amount of loaded calcein in the nanodroplets was measured by freezing 200 μL of the sample overnight in a −80 °C freezer, followed by lyophilization (Labconco, MO, USA) for 1 day. The resulting powder was resuspended in 200 μL PBS and centrifuged at 4500 RCF for 3 min. The 100 μL resulting supernatant was added to a black 96-well plate. Using a microplate reader (Cytation 7; BioTek Instruments, VT, USA) with the standard plate reader software (Gen 5; BioTek Instruments, VT, USA), the fluorescence of each well was measured at an excitation wavelength of 485 nm and an emission wavelength of 528 nm. The calcein concentration was determined based on a standard.

To characterize the stability of the particle, sizing and released model drug measurements were conducted using the same procedure as before, at one, three, five, and 7 days after synthesis.

Cytotoxicity was quantified through an MTT assay. RAW 264.7 cells were seeded at 2 × 10^5^ cells per well in a 96-well plate. dePFCnDs were added to the cells on the following day at varying concentrations, with PBS serving as a control. On the subsequent day, MTT (5 mg/mL in PBS, 10 μL) was added to each well and incubated for 2 h. Afterward, the supernatant was removed, and the crystals were dissolved in DMSO (150 μL). The DMSO solution was allowed to settle to remove the influence of excess nanodroplets, and then the supernatant from each well (100 μL) was transferred to another well. The absorbance was measured at 590 nm using the microplate reader (Synergy HT; BioTek Instruments, VT, USA).

The vaporization threshold was determined by embedding the dePFCnDs within a polyacrylamide phantom, which was synthesized using the same methodology as previously described. In this case, dePFCnDs (400 μL) were added to the phantom to yield a 1:500 dilution of stock dePFCnDs. The phantom was then irradiated with multiple pulses of increasing fluence, ranging from 10 to 75 mJ/cm^2^, with three pulses per fluence, followed by 10 pulses at the peak fluence and then a ramp-down in fluence using the same fluences as the ramp-up cycle. After each pulse, five ultrafast B-mode US images were captured at a pulse repetition frequency of 2 kHz using 0° planar US waves. Differential frames were determined by subtracting the fifth frame from the first frame, and the vaporization threshold was determined by plotting the integrated differential US intensity against the fluence acquired during the ramp-down cycle, which was then fitted to a sigmoidal curve. The threshold was identified as the intersection of a line drawn from the midpoint of the curve to the *x*-axis, which shared the same slope as the midpoint of the curve.

### Measurements of Encapsulated Cargo Release Efficiency in dePFCnDs

To evaluate the release efficiency of nanodroplets under laser irradiation, 200 μL of 2-fold diluted dePFCnDs loaded with calcein were placed in each well of 96-well plates, and each well was irradiated by a custom-built well plate lasing system. The system consists of a 10 Hz pulsed Nd: YAG laser (Vibrant; Opotek Inc., CA, USA) with a 3-axis motion stage, and the number of pulses and lasing intensity irradiated into each well can be adjusted using LabVIEW software (National Instruments, TX, USA). To evaluate the effect of laser intensity on calcein release, the number of pulses was fixed at 100 pulses, and the laser intensity was varied to 0, 20, 50, 70, and 100 mJ/cm^2^. Similarly, to investigate the effect of laser intensity, the laser fluence was fixed at 70 mJ/cm^2^, and the number of pulses was varied to 0, 10, 100, and 1000.

To quantify the release efficiency of the lased dePFCnDs samples, the irradiated samples were centrifuged at 4500 RCF for 3 min to remove residual nanodroplets. The 100 μL of resulting supernatant was added to a black 96-well plate, and the concentration was determined as described in the method for measuring the loading efficiency of calcein. The release efficiency was then calculated by dividing the released calcein by the loaded calcein concentration.

To determine the effect of heating on release and size variation, the 2 times diluted dePFCnDs were placed in a mini heat block (Fisherbrand, MA, USA) at 25, 37.5, 50, 60, and 70 °C for 10 min. Dynamic light scattering (DLS, Zetasizer Nano ZS; Malvern Panalytical, Malvern, UK) was used to determine the size after heating. 200 μL of heated samples were transferred to a 1.5 mL microtube (USA Scientific, FL, USA) and centrifuged at 4500 RCF for 3 min using a mini-centrifuge (5452 minispin Centrifuge; Eppendorf, Hamburg, Germany) to remove residual nanodroplets. The 100 μL resulting supernatant was added to a black 96-well plate, and the concentration was determined as previously described.

### Poly I:C Encapsulated dePFCnDs

To determine whether the probe sonication process affects poly I:C fragmentation, we measured the amount of nitrite produced in RAW 264.7 cells after adding poly I:C, both without probe sonication and after two rounds of probe sonication required for dePFCnDs formation, using the Griess assay.

Agarose gel electrophoresis was used to visualize the release of poly I:C from the dePFCnD. A 1% agarose gel was cast by dissolving 1 g of agarose (Sigma-Aldrich) in 100 mL of TAE buffer (Tris-acetate-EDTA, ThermoFisher Scientific). The solution was then heated in a microwave until the agarose was fully dissolved. The solution was then poured into a mold and allowed to cool until the gel solution solidified. Poly I:C (2.5 μg), poly I:C loaded dePFCnD, and poly I:C loaded dePFCnD with FUS stimulation were loaded into separate wells with 6x gel loading dye (NEB, MA, USA), and the gel was run with TAE as a running buffer at 120 V at 0.05 A for 30 min. The resulting gel was then stained with SYBR Gold Nucleic Acid Gel Stain (ThermoFisher Scientific) for 10 min. The gel was then imaged using a Gel Doc EZ Imager (Bio-Rad, CA, USA).

The RAW 264.7 cells were seeded in a 24-well plate with a fluorocarbon film bottom (Boca Scientific, MA, USA), at a density of 1.2 × 10^6^ cells per well (1 mL) and incubated for 24 h. Following the 24 h incubation, the media was replaced with 1.8 mL of phenol-free DMEM. Subsequently, 180 μL of three different solutions (dePFCnDs loaded with poly I:C, 1 mg/mL concentration free poly I:C, and sterile PBS) were individually added to separate wells.

Following the addition of particle solutions to the media, the well plates were placed in a 37 °C water bath. Half of the wells underwent sonication for 5 min using a customized FUS system to deliver acoustic energy to a cell culture well plate. A 2 MHz transducer was placed at the bottom of the well plate to deliver acoustic energy from bottom to top. The FUS sonication was applied to each well with 1.08 MPa peak negative pressure, 50% duty cycle, and 15 ms pulse repetition interval. To prevent media from splashing onto other samples during sonication, the lid of the well plate was kept closed. After sonication, the 24-well plates were returned to the incubator.

After 24 h of incubation, 100 μL of supernatant was removed from each well and transferred to a well within a 96-well plate. The first three columns of this well plate were loaded with sodium nitrite at concentrations ranging from 25 to 0.39 and 0 μM dissolved in phenol-free DMEM solvent as a standard. A 40 mg/mL solution of modified Griess reagent (Sigma-Aldrich) was prepared in deionized water. Then, 100 μL of Griess reagent solution was added to each well of the 96-well plate.

After adding the Griess reagent to the samples, the 96-well plate was covered with aluminum foil to minimize light exposure. The plate was incubated for 10 min and the absorbance of each well was measured using a microplate reader (Synergy HT; BioTek Instruments, VT, USA) with the standard plate reader software (Gen 5; BioTek Instruments, VT, USA) at 546 nm. The absorbance data were converted to moles of nitrite using the standard.

### Focused Ultrasound Parameter Optimization for Encapsulated Cargo Release of dePFCnDs

To optimize the release effect of nanodroplets through FUS, dePFCnDs were suspended within a polyacrylamide cuvette phantom and exposed to a range of acoustic parameters.

The polyacrylamide cuvette phantom was synthesized by combining 50 mL of a 40% polyacrylamide solution (Sigma-Aldrich, MO, USA), 150 mL of deionized water, and 2 mL of a 10% ammonium sulfate solution (APS, Sigma-Aldrich, MO, USA). This mixture was placed in a vacuum flask, sealed with a rubber stopper, and connected to a vacuum. The solution was then degassed using a water bath sonicator. After degassing, 250 μL of TEMED (*N*,*N*,*N*′,*N*′-Tetramethylethylenediamine, Sigma-Aldrich, MO, USA) was added to the degassed solution and poured into a rectangular plastic mold to solidify. During the solidification process, a plastic cuvette was inserted into the center of the phantom to create a rectangular void. Once the phantom was fully polymerized, the cuvette was removed, and the phantom was extracted from the plastic mold.

The cuvette phantom was placed in a 37 °C water bath (WB-200; Cole-Parmer, IL, USA), and the dePFCnDs samples were diluted 2-fold and placed within the rectangular void area of the phantom. FUS sonication was performed using a ring-shaped single-element 2 MHz FUS transducer (H-148, Sonic Concept, WA, USA) with a focal length of 63.2 mm. The 2 MHz sinusoidal pulse with varying peak negative pressures (PNP), duty cycles, pulse repetition intervals (PRI), and durations were generated by a function generator (AFG3022C; Tektronix Inc., OR, USA). This pulse was amplified by 55 dB with an RF power amplifier (1040L; Electronic and Innovation, NY, USA) and transmitted via an impedance-matching circuit to the FUS transducer. To identify the optimal acoustic parameters for efficient release, individual ultrasound parameters were systematically varied while maintaining other parameters at their default values: PNP of 1.44 MPa, duty cycle of 50%, PRI of 300 ms, and sonication duration of 120 s. Specifically, for PNP optimization, values ranging from 0 to 2.16 MPa were tested at intervals of 0.36 MPa, and acoustic pressures were measured using a calibrated hydrophone immersed in degassed water. For duty cycle optimization, seven duty cycle conditions were evaluated (5, 10, 20, 25, 50, 80, and 100%). The sonication duration was tested by varying exposure times (0, 10, 30, 60, 120, 300, and 600 s) at three different PNP levels (1.08, 1.44, and 2.16 MPa). Lastly, the PRI was optimized by examining intervals of 1, 3, 5, 7, 10, 15, 20, 30, 40, 50, 100, and 300 ms at three distinct duty cycles (20, 50, and 80%). The release efficiency was quantified based on fluorescence, as stated earlier.

### Cavitation Signals of the dePFCnDs under FUS Sonication

To investigate structural changes of dePFCnDs, associated cavitation signals, and release kinetics, PBS or dePFCnDs were sonicated with FUS in a tube, and passive acoustic signals were recorded. This setup included a 500 kHz FUS transducer (Sonic Concepts, WA, USA) along with a passive cavitation detector (PCD) to receive acoustic signals. At the focus, the FUS transducer delivered a PNP of 1 MPa. The cavitation signals were acquired under four states: PBS during the FUS, dePFCnDs before laser activation during short-pulse (20 pulses) FUS, vaporized dePFCnDs after laser activation during short-pulse (20 pulses) FUS, and disrupted droplets generated by long-pulse (10,000 pulses) FUS after laser activation.

### Ultrasound and Photoacoustic Guided FUS System

The US/PA-guided FUS system comprised a Vantage 128 ultrasound research platform (Verasonics Inc., Kirkland, WA, USA), a 128-element linear transducer L11-4v (Verasonics Inc., WA, USA), an Nd: YAG pulsed laser Tempest (1064 nm; New wave Research, Fremont, CA, USA) and FUS system comprising a function generator, amplifier, matching circuit and FUS transducer and allowing to adjust parameter such as frequency, PNP, duty cycle, and PRI using host controller PC. The estimated fluence at the output of the fiber bundles, measured by a laser power meter (Nova II, Ophir-Spiricon, UT, USA), was 75 mJ/cm^2^.

B-mode images were taken at a pulse repetition frequency of 2 kHz using 0° planar US waves before and after laser irradiation, and the PA images were acquired immediately after each laser pulse. The differential US image (ΔUS) was obtained by subtracting the background US images obtained before laser irradiation from those obtained after laser irradiation.

### In vitro Encapsulated Cargo Release and US/PA Images

To visualize the dePFCnDs using US/PA imaging and monitor changes in US and PA signals after various degrees of encapsulated cargo release through FUS, a polyacrylamide tube phantom was used.

The polyacrylamide tube phantom was synthesized similarly to the cuvette phantom, except that a 5 mm diameter plastic pipet was utilized to create a tubular void. After focused ultrasound-induced model drug release with varying efficiencies, the dePFCnDs samples were diluted 500 times and placed in a tube within the phantom. Both ends of the tube were sealed with ultrasound gel. The phantom was imaged using PA and plane-wave US imaging. PA intensity and US intensity were determined by integrating over the region of interest.

### In vivo US/PA Images

In vivo US/PA imaging studies were performed under the approval of Georgia Tech Institutional Animal Care and Use Committee (protocol no. A100281). BALB/c mice (4–6 weeks old; The Jackson Laboratory, Bar Harbor, ME, USA) received a subcutaneous flank inoculation of 5 × 10^6^ 4T1 breast cancer cells (50 μL). Imaging began once tumor volumes reached at least 150 mm^3^. Baseline US/PA images were acquired before dosing, followed by intratumoral injection of dePFCnDs (50 μL, 10^9^ droplets/mL). To minimize needle-track artifacts and short-lived baseline drift and to allow dispersion, postinjection US/PA imaging was performed 10 min after the injection; this delay improves interpretability by reducing artifacts unrelated to dePFCnDs. FUS stimulations were performed twice, with a duration of 300 s for each stimulation, 50% duty cycle, and 15 ms PRI. A 5 min interval separated the two FUS exposures to allow thermal relaxation, droplet recondensation, and recovery of baseline US/PA signals, thereby limiting cumulative heating and residual cavitation. To check the difference in the intensity of the FUS, experiments were conducted on low-intensity FUS (PNP 1.08 MPa) and high-intensity FUS (PNP 2.16 MPa), respectively. US/PA images were acquired immediately after each FUS exposure using the previously described imaging sequence. During high-intensity FUS, the imaging transducer operated in receive-only mode to record passive acoustic emissions, and cavitation maps were reconstructed by passive acoustic mapping to monitor dePFCnD activation and encapsulated cargo release.

## Data Availability

Data that supports the findings of this study are available from the corresponding author upon request.

## Abbreviations

dePFCnDs: double-emulsion perfluorocarbon nanodroplets
DLS: dynamic light scattering
FUS: focused ultrasound
PA: photoacoustic
PCD: passive cavitation detector
PFC: perfluorocarbon
PFCnDs: perfluorocarbon nanodroplets
PNP: peak negative pressure
poly I:C: polyinosinic-polycytidylic acid
PRI: pulse repetition interval
TAE: Tris-acetate-EDTA
TLR3: toll-like receptor3
TEM: transmission electron microscopy
US: ultrasound
